# Participatory research in child psychology & psychiatry: Embracing untidiness to break new ground

**DOI:** 10.1002/jcv2.12284

**Published:** 2024-12-04

**Authors:** Andrea MacLeod, Ifigeneia Manitsa, Stephane De Brito

**Affiliations:** ^1^ Autism Centre for Education and Research University of Birmingham Birmingham UK; ^2^ Department of Disability Inclusion and Special Needs University of Birmingham Birmingham UK; ^3^ Institute for Mental Health and Department of Disability Inclusion and Special Needs (DISN) University of Birmingham Birmingham UK; ^4^ Centre for Human Brain Health University of Birmingham Birmingham UK; ^5^ Institute for Mental Health University of Birmingham Birmingham UK; ^6^ Centre for Developmental Science School of Psychology University of Birmingham Birmingham UK; ^7^ Centre for Neurogenetics University of Birmingham Birmingham UK

## Abstract

We are pleased to present our Special Issue on Participatory Research. In this editorial, we outline how the disability rights movement has been instrumental to the development of participatory approaches, before giving consideration to some of the debates and criticisms associated with participatory research in practice. We summarise the contributions offered by the studies within this issue and propose four areas of consideration, drawn from the body of included research, to inform future developments.

## CONTEXTUALISING PARTICIPATORY RESEARCH

Participatory research offers the potential to transform our understanding of complex phenomena by situating it in the experiences of those affected—it provides an opportunity for ‘a new look at old questions’ (Jafee, 2020, p. 215). Although participatory approaches have been around in one form or another for some years, they have gained traction relatively recently. Originally linked to the disability movement and advocated by disabled scholars as a means of advancing a more representative and relevant research agenda (Oliver, 1992), they are now increasingly being valued by funders and policy makers as a means of increasing the relevance of a wide array of human research. Community based participatory research (CBPR) in the US/Canada and Patient & Public Involvement (PPI) in the UK are two examples. Meaningful and active stakeholder involvement is now often deemed essential in research proposals by primary funders such as the National Institute for Health and Care Research (NIHR) and the UNCRC (1989) has been deemed influential to the growth of participatory research with children (Horgan, 2017). There is growing evidence that participatory research in mental health can be valuable in evidencing interventions more suited to user needs, but also a need to better understand both what it can offer and where it can be problematic (Güell et al., 2023).

## DILEMMAS & DEBATES

There are a number of tensions and limitations worthy of consideration by way of introducing this special issue. Assumptions can be made about representativeness, so that certain perspectives (biased towards the kinds of people more likely to volunteer) are highlighted at the expense of those who may be less willing or able to get involved (Kitchin, 2000). Participatory research often employs creative methods and tends to require a greater time commitment than more traditional methods, which may not reflect participants' priorities so much as those of the researchers (Bourke, 2009). Moreover, a greater investment of time by participants can inadvertently lead to specific ethical risks in relation to vulnerable populations, who might be more socially isolated and therefore hope for a more long‐term connection than is available.

An implicit goal of participatory research is to disrupt the traditional researcher: subject power dynamic. However, in considering research with children, there are some specific challenges to navigate. Adult gatekeepers will always be part of the process to some degree, whether they be researchers, teachers or parents. This can lead to tensions between, for example, child autonomy and parental consent (Horgan, 2017). Whilst intentions may be good, it is possible that existing power dynamics are simply reinforced by the structures through which the research takes place, or even cause new problems. Research that takes place in schools or clinical settings is affected by the nature of their staff and infrastructure in ways that cannot always be predicted or managed. Additionally, co‐researchers may be overly influenced by the values and views of the lead researchers or may have different interpretations of fundamental ethical principles such as confidentiality (Nind, 2014).

Loveridge et al. sum up these dilemmas nicely when they suggest that researchers must accept the untidiness of participatory research (2024). Participatory researchers should reflect upon their own positionality and engage with ‘every‐day ethical moments’ rather than relying on formal ethical safeguards (Loveridge et al., 2024, p. 405). In the studies shared within this issue, we can observe researchers doing just this.

Our sister journal, *The Journal of Child Psychology and Psychiatry*, recently underlined the benefits of using participatory research in the development of clinical interventions for vulnerable populations such as individuals with ADHD (Agnew‐Blais & Michelini, 2023) and autism (Pellicano, 2020). Additionally, it has published a range of examples of participatory research, applying methodologies that involve both discovery and translation research, such as literature reviews, inductive thematic context analysis and intervention development (e.g., Foster et al., 2024; Williams et al., 2023).

Such studies have set the stage for the current interdisciplinary special issue focussing on the use of participatory research methods in the fields of mental health, child and adolescent development, and neurodevelopmental conditions.

The empirical research reported in this issue represents a novel range of topics as well as collaborators from different age groups. These provide important contributions to the ongoing dialogue about how participatory research can best offer research that is ethical to its stakeholders, meaningful to its communities and possesses the rigour necessary to advance knowledge.

## INTRODUCING OUR SPECIAL ISSUE PAPERS

Khawaja et al. (2024) consider the methodological challenges and potential benefits of facilitating youth participatory action research (YPAR) within school settings, contextualising this within its origins, aims and epistemological principles. The authors have addressed a common criticism—that participatory research can sometimes be presented as straightforward and unproblematic, with methodological detail that is descriptive rather than reflective. The authors overtly avoided this, reflecting in depth on the ethical dilemmas and power imbalances that can come into play when young people work within an adult‐led team, on a project bound by specific timelines and objectives. They reasoned that knowledge production in this kind of context may not achieve an ‘idealized democracy’. However, they argued persuasively that researcher self‐awareness and openness about tensions, and even failures, could and should form a vital part of the story. Encouragingly, some youth participants from their project went on to sit on other working groups as a result of their involvement, illustrating the ways in which such projects can create impact that go beyond their original intentions or expectations.

Bartnick et al. (2024) report on the foundation of a ‘Children's Council’ in Germany, a significant innovation focussing on primary aged children aged 6–9. Their work offers important insights into the development of child‐friendly research topics for mental health disorders and prevention and develops guidelines for advisory research groups with young children. The authors deliberately recruited through a Mental Health service in order to ensure relevant personal experience and reflect on the need for children and young people to have ‘mental health literacy’. Their account of the practical challenges and additional ethical considerations in research with young children throws valuable light on an area which is often avoided due to its inherent complexities. Equally, their description of the ways in which the child collaborators advised on these aspects is itself a powerful indicator of the insights to be gained from this type of approach and the ‘mutual learning’ that can take place.

Babbage et al. (2024) focus on the development of a digital application to reduce self‐harm in young people. This interdisciplinary project focussed on young and included eleven co‐researchers with lived experience of self‐harm. The authors describe a conscious investment of time to recruit a diverse group and they reflect on the need to ‘sit with a level of risk’ in research of this kind. This kind of cost‐benefit analysis should form part of the ethical practice of any research, but having it articulated in this way is especially valuable for research which aims to address sensitive topics and/or vulnerable populations, because it underlines the critical fact that if done mindfully, the benefits can—and do—outweigh the risks. This recognition serves to ensure that such populations are not inadvertently silenced by the avoidance of research through a misguided fear of harm.

Two papers focus on neurodivergence and employed a two‐tier method, whereby older (therefore perhaps more experienced and confident) researchers undertook some initial work which informed later research with younger participants. McKinney et al. (2024) set out to explore camouflaging in neurodivergent adolescent girls. The research priority of camouflaging was directly informed by the (adult) neurodivergent co‐production team, which then informed the focus and design of a relatively large‐scale study with a younger group of participants. The study identifies an important link between camouflaging and poor mental health in early adolescence and employs a transdiagnostic approach. As Crane (2024) considers in her commentary, this is increasingly valued within research and interventions related to neurodivergence due to the high rates of co‐occurring conditions and overlapping characteristics. Kakoulidou et al. (2024) share their work in the co‐development of a framework to involve neurodivergent participants in translational research. Their Youth Research Panel were aged 18–25, working with participants aged 11–15 to develop a protocol for the co‐production of participatory qualitative research, including data coding and analysis.

Both studies employed a mixed‐methods approach and more experienced ‘peer’ co‐researchers. They offer exemplars for balancing three critical but complex components of successful participatory research: the need to have meaningful involvement from an early stage of the research, to consult as widely as possible without having unreasonable expectations of participants (or indeed, research costs) *and* to produce research that demonstrates sufficient rigour to make a contribution to the field. Although in these studies, the youngest co‐researchers were aged 11, this might offer a useful model for work involving younger children, since it potentially minimises demands and maximises sensitivity to their needs and priorities.

In their review of 50 studies, Bakermans‐Kranenburg and van IJzendoorn (2024) critically examine the role and impact of PPI in studies on youth mental health. They evaluate whether the involvement of young participants and other stakeholders enhances the relevance and applicability of research findings, cautioning against prematurely translating exploratory PPI findings into clinical or policy recommendations without robust evidence and replication. Whilst PPI is praised for encouraging the generation of hypotheses and incorporating diverse perspectives, significant issues with transparency and replicability remain, raising concerns about the responsible use of PPI‐driven findings. Finally, the review discusses the complexities of awarding co‐authorship to PPI participants, noting that while their involvement is valuable, it may not align with current scientific authorship guidelines. The authors rightly call for clearer standards to ensure ethical and effective use of PPI in research.

In their rapid realise review based on 57 papers, Jones et al. (2024) aim to build a better understanding of the effectiveness of co‐production in youth mental health services by investigating for whom and in what contexts co‐production works. The authors usefully organised their findings using Context–Mechanism–Outcome (CMO) configurations, highlighting that effective co‐production requires a supportive organisational culture, transparency about limitations, and authentic power‐sharing between young people and service providers. The authors note that when these conditions are met, youth, especially from underrepresented communities, can engage meaningfully and feel their knowledge is valued. Crucially, this often leads to personal development gains—such as improved confidence and professional skills—while also enhancing service quality and relevance through their input. However, Jones and colleagues highlight the importance of avoiding tokenism and ensuring youth voices contribute to tangible, rather than symbolic involvement.

## CONCLUSIONS AND FUTURE DIRECTIONS

The peer‐reviewed papers included in this special issue offer novel evidence‐based guidance on the active involvement of children and young people in research. In common with other participatory research studies, authors have reflected on the need for creative approaches, sensitivity to participants' life experiences and enough time to build the knowledge and trust required for participants to contribute meaningfully and with confidence. Perhaps not surprisingly, they all report that these investments paid off. Most importantly, they provide detailed, transparent evidence of both how this was achieved, and where it did not. There is much here from which we can learn. In many cases, the studies included detailed recommendations specific to their context. Therefore rather than repeat these, we summarise below key points from this body of work as a whole.

We encourage participatory researchers in our field to consider that:

- Research outcomes and processes are best served by a willingness to be open about the challenges and to reflect on positionality, not just of the research team but of everyone involved
- Ability to actively participate may depend more on the creativity and flexibility of the approach and team than on the age or profile of the participant
- Projects which incorporate older and younger stakeholders in their design can combine different kinds of participatory input towards a common goal
- Sitting with a level of risk may be an essential part of participatory research in this field, but if this is undertaken with sufficient planning and reflection, the mutual learning can offer insights and outcomes that would not otherwise be gained.

## AUTHOR CONTRIBUTIONS

**Andrea MacLeod**: Conceptualization; writing—original draft; writing—review & editing. **Ifigeneia Manitsa**: Writing—original draft; writing—review and editing. **Stephane De Brito**: Writing—original draft; writing—review and editing.

## CONFLICT OF INTEREST STATEMENT

Andrea MacLeod and Ifigeneia Manitsa declare no conflict of interests; Stephane De Brito is one the Joint Editors of *JCPP Advances*.

## ETHICAL CONSIDERATIONS

None.

## Data Availability

Data sharing not applicable to this article as no datasets were generated or analysed during the current study.
